# Supplementary material for: In vivo Firre and Dxz4 deletion elucidates roles for autosomal gene regulation
Source: eLife. 2019 Nov 18;8:e47214. doi: 10.7554/eLife.47214 (PMC6860989; doi:10.7554/eLife.47214)
Supplement: Transparent reporting form [file elife-47214-transrepform.pdf]

eLIFE
1st Floor
24 Hills Road
Cambridge CB2 1JP, UK
P 01223 855340
W elifesciences.org
T @elife

eLife's transparent reporting form

We encourage authors to provide detailed information *within their submission* to facilitate the interpretation and replication of experiments. Authors can upload supporting documentation to indicate the use of appropriate reporting guidelines for health-related research (see EQUATOR Network), life science research (see the BioSharing Information Resource), or the ARRIVE guidelines for reporting work involving animal research. Where applicable, authors should refer to any relevant reporting standards documents in this form.

If you have any questions, please consult our Journal Policies and/or contact us: editorial@elifesciences.org.

Sample-size estimation

- You should state whether an appropriate sample size was computed when the study was being designed
- You should state the statistical method of sample size computation and any required assumptions
- If no explicit power analysis was used, you should describe how you decided what sample (replicate) size (number) to use

Please outline where this information can be found within the submission (e.g., sections or figure legends), or explain why this information doesn't apply to your submission:

We used at least three biological replicates for each group (3WT vs 3KO). This number of replicates is generally used for RNA-seq in tissues collected from inbred mice, since a low biological and technical variability among the samples is expected. To test the variability in our samples we performed unsupervised clustering (Pearson correlation) and were able to confirm the identity of the tissues, by showing that replicates of the same tissue clustered together. This, result implies a low biological and technical variability across the samples which supports our choice of at least 3 biological replicates.

In addition, for validation of the dysregulated genes observed in the double deletion we use independently generated single KO mouse strains (2 or 3 replicates per group).

Replicates

- You should report how often each experiment was performed
- You should include a definition of biological versus technical replication
- The data obtained should be provided and sufficient information should be provided to indicate the number of independent biological and/or technical replicates
- If you encountered any outliers, you should describe how these were handled
- Criteria for exclusion/inclusion of data should be clearly stated
- High-throughput sequence data should be uploaded before submission, with a private link for reviewers provided (these are available from both GEO and ArrayExpress)

Please outline where this information can be found within the submission (e.g., sections or figure legends), or explain why this information doesn't apply to your submission:

eLife Sciences Publications, Ltd is a limited liability non-profit non-stock corporation incorporated in the State of Delaware, USA, with company number 5030732, and is registered in the UK with company number FC030576 and branch number BR015634 at the address 1st Floor, 24 Hills Road, Cambridge CB2 1JP | August 2014

Each RNA-seq experiment was performed once. In the manuscript we use biological replicates, defined as tissue derived from different individuals, as detailed in the Figures, Figure legends and Material and Methods. We did not conduct technical replicates in this study.

To determine whether the deletion of *Firre*, *Dxz4* or *Firre-Dxz4* impact random or imprinted X chromosome inactivation, we collected embryonic day 12.5 brains, placentas and visceral yolk sac from reciprocal F1 crosses between the deletion strains and CAST/EiJ. For the brain and the placenta, we collected samples for all three strains from the forward cross (3 males and 3 females for WT and maternal deletion) and reverse cross (3 females for WT and paternal deletion). For the placenta DKO we added an additional replicate of the maternal and paternal deletion. In addition, we collected visceral yolk sac samples from the DKO forward cross (3 females WT and maternal deletion) and reverse cross (3 females WT and paternal deletion).

Moreover, to test whether DNA methylation levels are altered in the absence of *Firre* and *Dxz4 on* either Xa *or* Xi, we collected placentas from the DKO forward cross (1 female WT and 2 maternal deletion) and reverse cross (2 females WT and 2 paternal deletion) to perform reduced representation bisulfite sequencing (RRBS) sequencing.

For the DKO adult bodymap, we collected the spleen, brain, kidney, heart, lung and liver from 6 weeks old female mice carrying a homozygous double deletion (4 WT and 4 DKO replicates). To validate and classify the dysregulated genes form the DKO, we collected liver and spleen from independent generated female SKO strains (*Firre* 3 SKO and *Dxz4* 2 SKO replicates). To test whether the molecular phenotype observed in the female spleen can be uncoupled form X inactivation, we collected 6 weeks old spleens from DKO males (2 WT and 2 DKO replicates).

We displayed outliers in the boxplot figures.

Sequence data and alignments have been submitted to the Gene Expression Omnibus (GEO) database under accession code GSE127554.

eLife Sciences Publications, Ltd is a limited liability non-profit non-stock corporation incorporated in the State of Delaware, USA, with company number 5030732, and is registered in the UK with company number FC030576 and branch number BR015634 at the address 1st Floor, 24 Hills Road, Cambridge CB2 1JP | August 2014

Statistical reporting

- Statistical analysis methods should be described and justified
- Raw data should be presented in figures whenever informative to do so (typically when N per group is less than 10)
- For each experiment, you should identify the statistical tests used, exact values of N, definitions of center, methods of multiple test correction, and dispersion and precision measures (e.g., mean, median, SD, SEM, confidence intervals; and, for the major substantive results, a measure of effect size (e.g., Pearson's r, Cohen's d)
- Report exact p-values wherever possible alongside the summary statistics and 95% confidence intervals. These should be reported for all key questions and not only when the p-value is less than 0.05.

Please outline where this information can be found within the submission (e.g., sections or figure legends), or explain why this information doesn't apply to your submission:

Statistical methods used in this study are described in the Figures, Figure legends and Material and Methods.

(For large datasets, or papers with a very large number of statistical tests, you may upload a single table file with tests, Ns, etc., with reference to sections in the manuscript.)

Group allocation

- Indicate how samples were allocated into experimental groups (in the case of clinical studies, please specify allocation to treatment method); if randomization was used, please also state if restricted randomization was applied
- Indicate if masking was used during group allocation, data collection and/or data analysis

Please outline where this information can be found within the submission (e.g., sections or figure legends), or explain why this information doesn't apply to your submission:

Groups were allocated according to tissue and genotype. The specific grouping for each figure is indicated in the corresponding Figure and Figure legend.

Additional data files ("source data")

- We encourage you to upload relevant additional data files, such as numerical data that are represented as a graph in a figure, or as a summary table
- Where provided, these should be in the most useful format, and they can be uploaded as "Source data" files linked to a main figure or table
- Include model definition files including the full list of parameters used
- Include code used for data analysis (e.g., R, MatLab)
- Avoid stating that data files are "available upon request"

Please indicate the figures or tables for which source data files have been provided:

eLife Sciences Publications, Ltd is a limited liability non-profit non-stock corporation incorporated in the State of Delaware, USA, with company number 5030732, and is registered in the UK with company number FC030576 and branch number BR015634 at the address 1st Floor, 24 Hills Road, Cambridge CB2 1JP | August 2014

eLIFE
1st Floor
24 Hills Road
Cambridge CB2 1JP, UK
P 01223 855340
W elifesciences.org
T @elife

Source data has been provided, including the processed data and R scripts for the figure generation.

Sequence data and alignments have been submitted to the Gene Expression Omnibus (GEO) database under accession code GSE127554.
To review GEO accession GSE127554:
Go to https://www.ncbi.nlm.nih.gov/geo/query/acc.cgi?acc=GSE127554
Enter token mfcvkggovjujfkt into the box
